# Adipose Tissue Distribution Predicts Survival in Amyotrophic Lateral Sclerosis

**DOI:** 10.1371/journal.pone.0067783

**Published:** 2013-06-27

**Authors:** Eva Lindauer, Luc Dupuis, Hans-Peter Müller, Heiko Neumann, Albert C. Ludolph, Jan Kassubek

**Affiliations:** 1 Department of Neurology, University of Ulm, Ulm, Germany; 2 Inserm U1118, Mécanismes centraux et périphériques de la neurodégénérescence, Strasbourg, France; 3 Université de Strasbourg, Faculté de Médecine, Strasbourg, France; 4 Institute of Neural Information Processing, University of Ulm, Ulm, Germany (HN); Banner Alzheimer’s Institute, United States of America

## Abstract

**Background:**

amyotrophic lateral sclerosis (ALS) is a neurodegenerative disease that leads to death within a few years after diagnosis. Malnutrition and weight loss are frequent and are indexes of poor prognosis. Total body fat and fat distribution have not been studied in ALS patients.

**Objectives:**

Our aim was to describe adipose tissue content and distribution in ALS patients.

**Design:**

We performed a cross-sectional study in a group of ALS patients (n = 62, mean disease duration 22 months) along with age and gender matched healthy controls (n = 62) using a MRI-based method to study quantitatively the fat distribution.

**Results:**

Total body fat of ALS patients was not changed as compared with controls. However, ALS patients displayed increased visceral fat and an increased ratio of visceral to subcutaneous fat. Visceral fat was not correlated with clinical severity as judged using the ALS functional rating scale (ALS-FRS-R), while subcutaneous fat in ALS patients correlated positively with ALS-FRS-R and disease progression. Multiple regression analysis showed that gender and ALS-FRS-R, but not site of onset, were significant predictors of total and subcutaneous fat. Increased subcutaneous fat predicted survival in male patients but not in female patients (p<0.05).

**Conclusions:**

Fat distribution is altered in ALS patients, with increased visceral fat as compared with healthy controls. Subcutaneous fat content is a predictor of survival of ALS patients.

## Introduction

Amyotrophic lateral sclerosis (ALS) is the most frequent adult-onset neurodegenerative motor neuron disorder (MND), with an incidence of 2–3/100 000, similar to that of multiple sclerosis. ALS is characterized by the simultaneous degeneration of bulbar and spinal motor neurons and of cortical upper motor neurons. ALS leads to death within 2 to 5 years of onset through failure of the respiratory muscles. There are currently no treatment for ALS except riluzole that prolongs lifespan for a few months.

Multiple evidence indicate that ALS is associated with abnormalities in nutritional status. [1] First, ALS patients lose weight throughout disease course, and this weight loss is a strong predictor of shortened survival. [2] Conversely, higher initial body mass index (BMI), [3], [4], [5] occurence of diabetes, [6] increased lipemia [7], [8] have been associated with prolonged survival in ALS patients. Our recent studies also showed that hepatic steatosis [7] and glucose intolerance [9] were unexpectedly observed in ALS patients despite weight loss. Most recently, results from the EPIC cohort showed that pre-diagnostic increased body fat was associated with decreased risk of ALS. [10] In animal models, increasing the lipid content of the diet offers neuroprotection and extends survival suggesting that nutritional interventions might slow down the disease process. [11], [12].

Adipose tissue stores constitute the major lipid stores in mammals. Studies of adipose tissue in ALS patients have been limited to global analysis of fat mass using bio-impedance metry. [2], [13] However, the topographical distribution of adipose tissues is of critical importance. Indeed, increased visceral fat is associated wth type 2 diabetes, dyslipidemia and insulin resistance while increased subcutaneous fat is associated with lower risk of metabolic syndrome and of diabetes, dyslipidemia and atherosclerosis. [14], [15], [16], [17] These associations are functionally relevant since transplantation of subcutaneous, but not visceral, adipose tissue exerts beneficial effects on energy metabolism in rodents. [18] Here, we studied fat distribution in a cohort of ALS patients and age and gender matched healthy controls using MRI based methods [19].

## Results

### Validation of ARTIS as a Measure of Total Fat in Humans

We measured fat content (FC_MRI_) in MRI from knees to diaphragm in 62 ALS patients and 62 healthy controls (baseline characteristics of both groups are shown in **Table 1**). Total fat as measured by the ARTIS algorithm was strongly correlated with BMI (**Supplementary** **Figure 1A**) (Spearman r = 0.647, p<0.0001) and fat content as determined using bioelectrical impedance measurement (**Supplementary** **Figure 1B**) (Spearman r = 0.646; p<0.0001). Circulating leptin levels are considered to be a reliable marker of body fat. Leptin serum levels were available in 28 patients. Indeed, we observed a strong correlation between leptin levels and FC_MRI_ (**Supplementary** **Figure 1C**) (Spearman r = 0.664; p<0.0001). These results indicate that MRI is a valuable tool to measure reliably body fat content.

**Figure 1:**
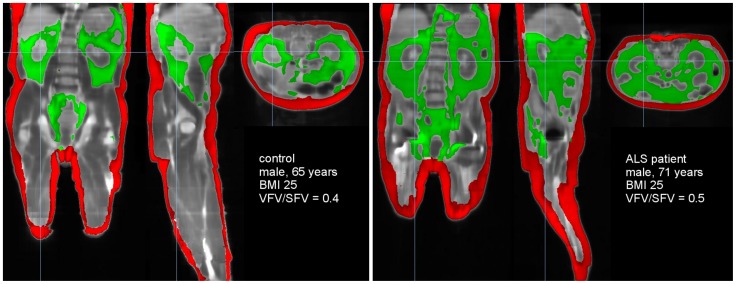
Comparison of fat deposits of an ALS patient and a control. Fat volume determination in a control (BMI = 25 kg/m^2^) (left) and in an ALS patient (BMI = 25 kg/m^2^) (right). Subcutaneous fat tissue is coloured in red, visceral fat tissue is coloured in green.

**Table 1:** Characteristics of patients and controls.

item	controls	ALS	p-value
number included	62	62	
M/F	36/26	42/20	0.3527
age (years)	59.9±12.2	59.9±12.1	1.000
BMI (kg/m^2^)	25.0±6.0	23.1±3.6	0.0004
site of onset (bulbar/spinal)		44 spinal	
		10 bulbar	
		7 unknown	
disease duration (months)		22.5±15.3	
ALS-FRS-R		36.3±7.5	

### Visceral Fat Accumulation is Increased in ALS

**Figure 2:**
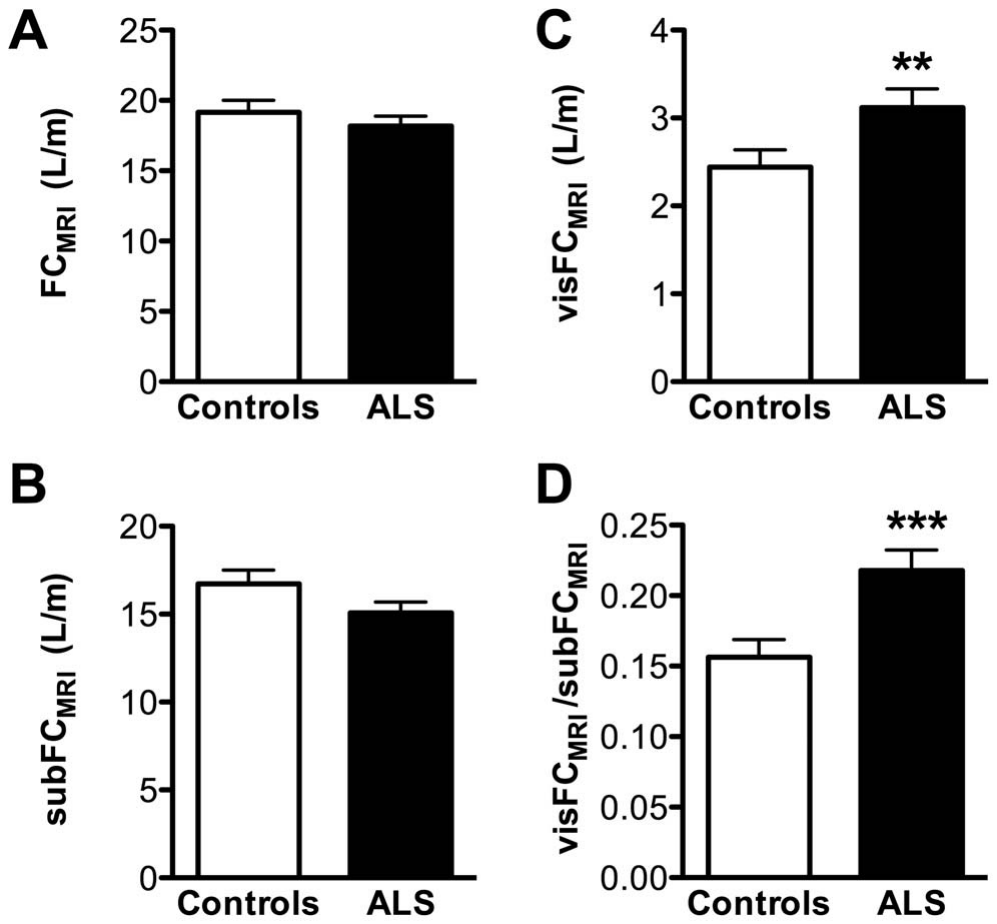
Topography of fat is altered in ALS. Total (FC_MRI_, A), subcutaneous (subFC_MRI_, B) and visceral (visFC_MRI_, C) fat content in MRI scans of controls (empty columns) and ALS patients (black columns). Panel D shows the ratio between visceral and subcutaneous fat. **, p<0.01, Mann-Whitney.

Contrasting with bioelectrical impedance measurement or biochemical markers, MRI allows to study the regional topography of the different fat pads. ALS patients displayed visibly expanded visceral fat deposits, and similar subcutaneous adipose tissue, as illustrated in **Figure 1**. Consistently, quantitative analysis showed that FC_MRI_ and subcutaneous fat content (subFC_MRI_) were roughly identical between both groups (p = 0.41 for FC_MRI_ and p = 0.09 for subFC_MRI_, **Figure 2A–B**), but visceral fat volume was increased in ALS patients (visFC_MRI_, **Figure 2C**, p = 0.0083). Thus, the ratio between visceral and subcutaneous fat pads was higher in ALS patients (**Figure 2D**, p = 0.001).

### Subcutaneous Fat Correlates with ALS-FRS-R

**Figure 3:**
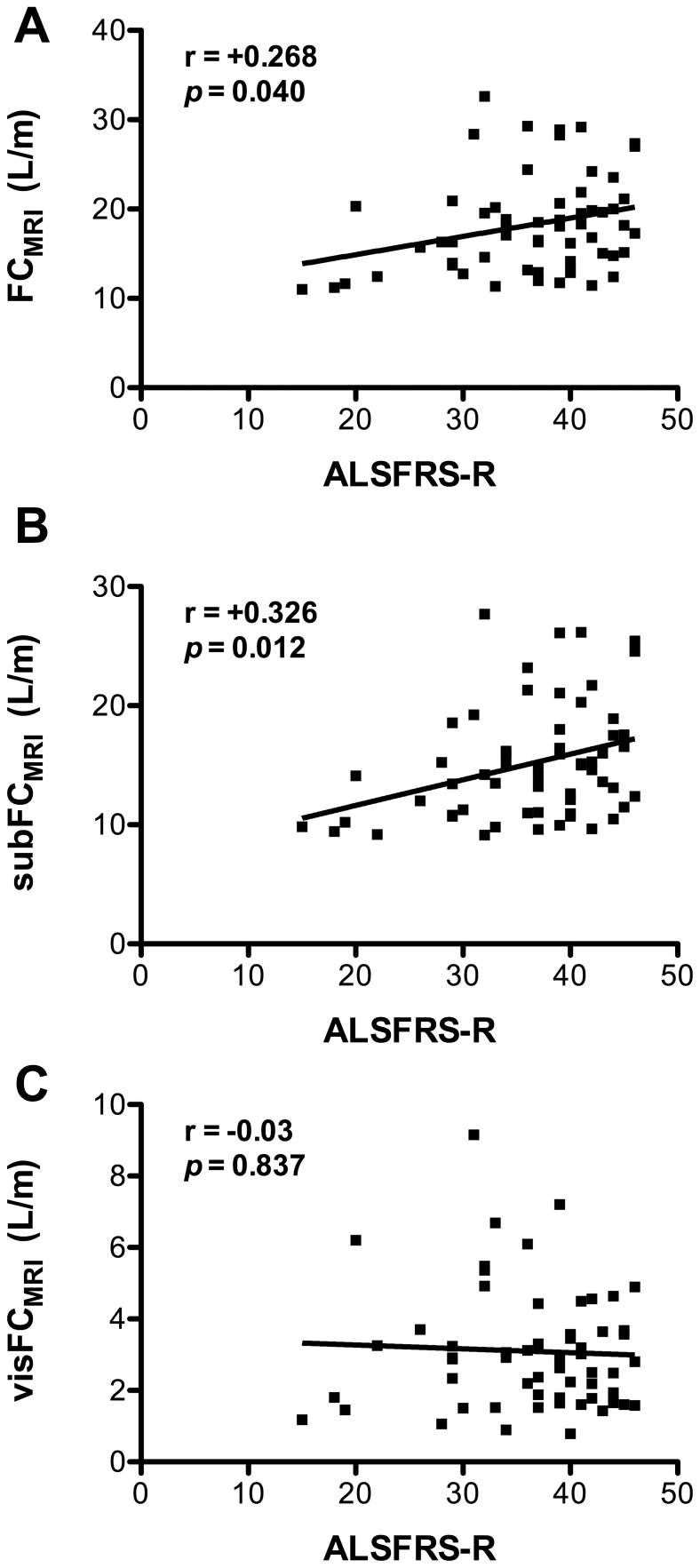
Subcutaneous fat content correlates with functional status of ALS patients. Correlations among Total (FC_MRI_, A), subcutaneous (subFC_MRI_, B), visceral (visFC_MRI_, C) fat content in MRI scans and amyotrophic lateral sclerosis functional rating scale scores (ALS-FRS-R). *p* values and the corresponding correlation coefficients (*r*) are indicated. If the correlation coefficient is positive, the two variables tend to increase or decrease together.

**Figure 4:**
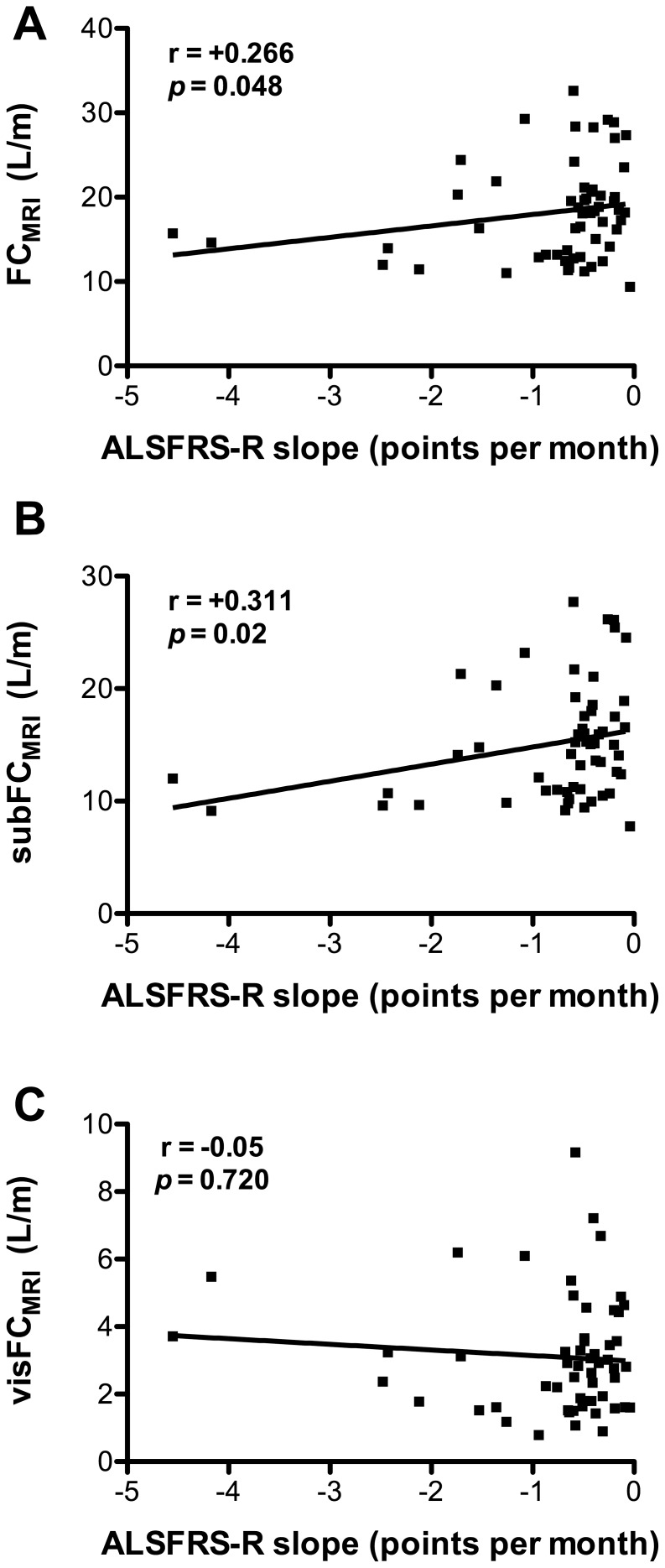
Subcutaneous fat content correlates with disease progression of ALS patients. Correlations among total (FC_MRI_, A), subcutaneous (subFC_MRI_, B), visceral (visFC_MRI_, C) fat content in MRI scans and slopes of amyotrophic lateral sclerosis functional rating scale scores (ALS-FRS-R, in points per month). *p* values and the corresponding correlation coefficients (*r*) are indicated. If the correlation coefficient is positive, the two variables tend to increase or decrease together.

**Figure 5:**
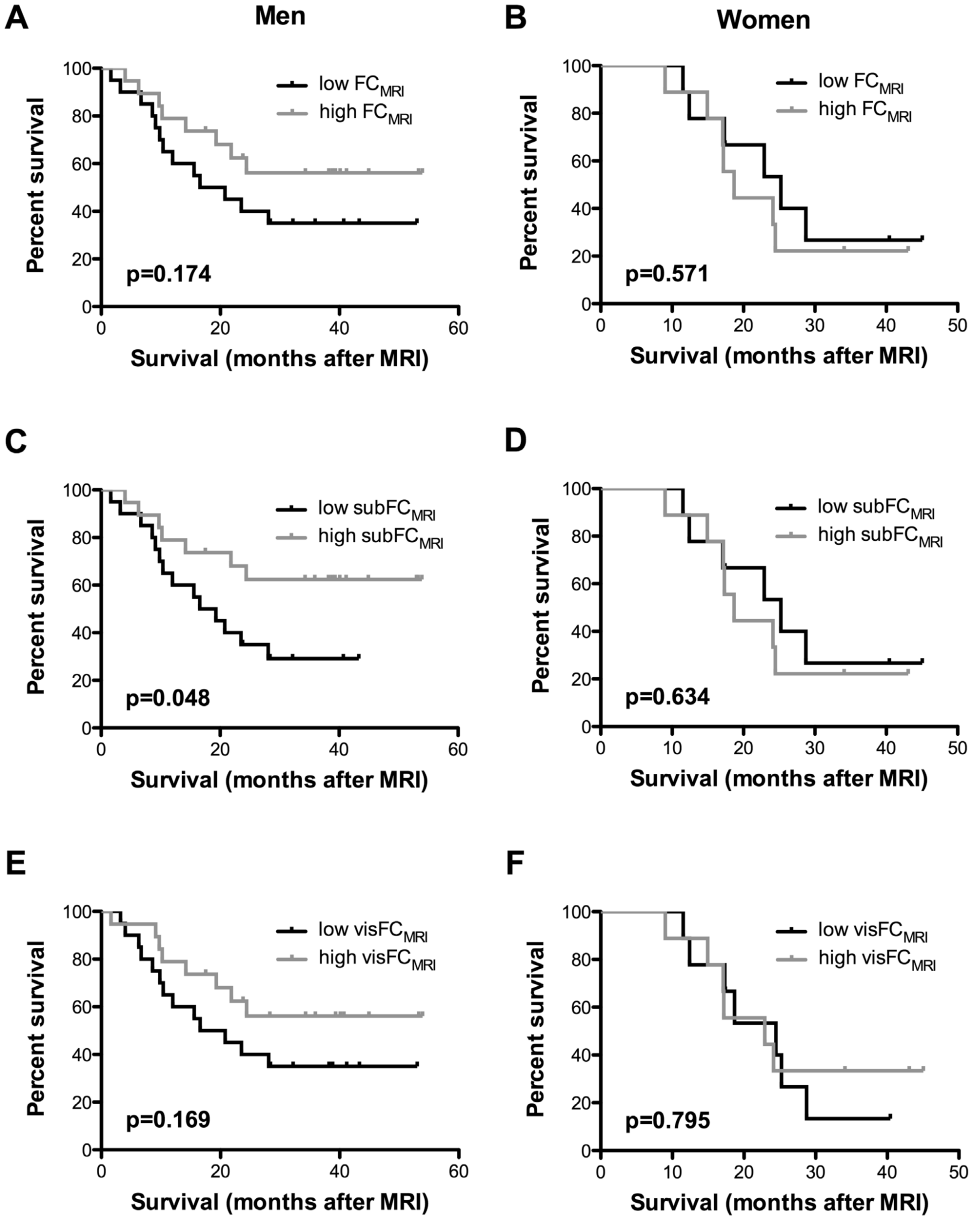
Kaplan-Meier survival curves stratified by gender and fat content. Male patients (A, C, E) and female patients (B, D, F) were stratified according to their total (FC_MRI_, A, B), subcutaneous (subFC_MRI_, C, D), and visceral (visFC_MRI_, E, F) fat content in MRI scans. *p* values (log-rank) are indicated. The black line represents patients below the median of the group; the grey line represents patients above the median of the group.

ALS-FRS-R score at the time of MRI correlated significantly with FC_MRI_ (r = 0.268, p = 0.04, **Figure 3A**), and this was due to subcutaneous fat since subFC_MRI_ correlated significantly with ALS-FRS (r = 0.326, p = 0.01, **Figure 3B**) whereas visFC_MRI_ did not (r = −0.03, p = 0.837, Figure 3C). Disease duration did correlate neither with FC_MRI_ (r = 0.001; p = 0.99) nor with visFC_MRI_ (r = 0.06; p = 0.66) nor with subFC_MRI_ (r = 0.01; p = 0.94). Disease progression, calculated as (ALS-FRS maximal score (i.e., 48) – ALS-FRS score at MRI)/disease duration correlated significantly with FC_MRI_ (r = 0.266, p = 0.048, **Figure 4A**). This was due to subcutaneous fat since subFC_MRI_ correlated significantly with disease progression (r = 0.311, p = 0.01, **Figure 4B**), in contrast to visFC_MRI_ (r = 0.05, p = 0.720, **Figure 4C**). It should however be noted that disease progression was very homogenous across patients, with most patients losing less than 1 point of ALS-FRS-R score per month. Fat distribution is likely to be modified by multiple factors, including gender, age, or MND-specific factors. We constructed a multiple linear regression model 
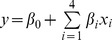
 for FC_MRI_, visFC_MRI_ and subFC_MRI_ including gender, age, site of onset and ALS-FRS-R. In these models, gender and ALS-FRS-R but not age or site of onset significantly attached to FC_MRI_ and subFC_MRI_. Gender, but not age, site of onset or ALS-FRS-R significantly attached to visFC_MRI_ (**Table 2**). Thus, functional status and disease progression of ALS patients correlated positively with subcutaneous fat. At the time of closure of our study (01/12/2012), 34 ALS patients had died and 23 were still alive. We had no information about the status of the five remaining patients who were excluded from the survival analysis. Since gender was a strong modifier of FC_MRI_ and subFC_MRI_, we performed survival analysis independently in male and female patients. Log-rank analysis indicated increased survival in male patients with higher subFC_MRI_ (p = 0.048), but not FC_MRI_ or visFC_MRI._(p = 0.174 and 0.169 respectively, **Figure 5A–C** **)**. This was not observed in female patients (p = 0.550, p = 0.634, and p = 0.795, respectively, **Figure 5C–E**). Thus, survival is predicted by subcutaneous fat content in a gender specific manner in ALS patients.

**Table 2:** Results of multiple linear regression models.

	FC_MRI_		visFC_MRI_		subFC_MRI_	
	*ß_i_*	p-value	*ß_i_*	p-value	*ß_i_*	p-value
constant (*ß_0_*)	10.775	0.092	1.967	0.294	8.808	0.088
ALS-FRS-R (*x_1_*)	**0.198**	**0.042**	0.012	0.668	**0.186**	**0.019**
gender (*x_2_*)	**3.117**	**0.049**	−**1.398**	**0.004**	**4.515**	**0.001**
age (*x_3_*)	0.025	0.685	0.030	0.099	−0.005	0.912
site of onset (*x_4_*)	−1.216	0.514	−0.379	0.492	−0.837	0.577

## Discussion

By use of automatic MRI bodyfat analysis, we showed that the distribution of fat is altered in ALS patients: Visceral fat was increased irrespective of disease severity, while subcutaneous fat was correlated with functional status and survival of ALS patients.

The first important result of our study is that fat distribution was not similar between ALS and healthy controls. While total fat content appeared roughly similar to controls, ALS patients displayed expanded visceral fat tissues and a trend towards decreased subcutaneous fat tissues. As a consequence, the ratio between visceral and subcutaneous fat increased. This situation appeared similar to what occurs in metabolic syndrome [14], [15], [16], [17] and is consistent with glucose intolerance [9] and hyperlipemia [7], [8] previously documented in other cohorts of patients. This abnormal fat distribution reminiscent of metabolic syndrome was paradoxically associated with weight loss and hypermetabolism. [1], [2], [3], . However, hypermetabolism and weight loss likely reflect increased lipid usage in the periphery. [23] Thus, ALS patients may display an uncoupling between lipid stores mobilization and metabolic/nutritional status. [1], [21] Of note, the fat distribution observed here in humans is strikingly different from animal models which, on the contrary, lose visceral fat through the course of the disease. [12].

Adiposity and its distribution reflect the whole body energy homeostasis, as the difference between energy intake and energy use. Neurodegeneration might impact adipose tissue through multiple mechanisms. First, increased visceral fat mass might be the consequence of inappropriate dietary intake. This appears to be the case in spinal cord injured (SCI) patients that prominently show increased visceral fat mass along with cardiovascular risk factors such as dyslipidemia. [24], [25], [26] In SCI patients, the combination of a dietary intake shifted towards increased fat consumption [27], [28] and of decreased energy expenditure [29], [30], [31] is likely to account for the modification of fat mass and distribution. This is, however, not the case in ALS since energy expenditure is increased and not decreased in these patients. [21], [32] Concerning food intake, eating habits of ALS patients have been poorly described. Although eating behavioural changes have been noted in some ALS patients [33] and in fronto-temporal dementia (FTD), a disease closely related to ALS, [34], [35] it remains unknown whether dietary intake is normal or not in these patients. A second mechanism that could account for increased visceral fat might be the degeneration of sympathetic nerve innervation of adipose tissue thereby precluding the CNS-induced mobilization of energy stores. [36] Indeed, multiple studies showed that sympathetic dysfunction is present to some extent in ALS patients. [37], [38] Whether this sympathetic dysfunction is present in adipose tissue is however unknown. Last, a change in distribution in fat mass might reflect an intrinsic defect in adipocytes. This appears to be the case in Huntington’s disease, at least in animal models. [39], [40], [41] Furthermore, in HD patients, leptin secretion (correlated with fat tissues) is correlated with CAG repeat number. [42] Interestingly, like ALS patients, HD patients display increased energy expenditure [43] and weight loss. Further work should determine whether adipocytes of ALS patients are intrinsically abnormal in their response to insulin and norepinephrine, or whether the defective lipid mobilization is due to degeneration/dysfunction of sympathetic innervation of adipose tissue.

We further showed that fat deposits of ALS patients displayed differential correlation with clinical parameters. While the amount of visceral fat was not correlated with functional status, we observed a strong positive correlation between subcutaneous tissue, functional status and survival. Recent work indicated that BMI was an independent predictor of survival in ALS, [2], [5] and dyslipidemia, also associated with longer survival, [7] is a surrogate marker of increased BMI. Interestingly, morbidly obese ALS patients display paradoxically decreased survival. Morbid obesity is strongly associated with increased visceral fat, and based on our results one might speculate that the development of morbid obesity, expanding visceral fat to the expense of subcutaneous fat, might have deleterious consequences on functional status and survival of ALS patients. Supporting this latter hypothesis, we observed increased survival in male patients with higher subcutaneous but not visceral fat (**Figure 5**).

Our current work is limited by it cross-sectional design. At the closure of our database, 24 months after the completion of MRIs, 57% of ALS patients had died. We observed improved survival in male patients with increased subcutaneous fat. Future work, with increased number of patients and longer follow-up, should be performed to confirm this first study. Furthermore, our cross-sectional design did not allow to monitor the evolution of fat distribution with disease progression in a given patient. Such information might also be of interest for the development of prognosis factors based on fat distribution. Another limitation of our current study is the homogenous disease progression in our patients cohort, which weakens the observed correlation between disease progression and fat distribution. Studies investigating fat distribution in fast versus slow progressors are thus warranted.

As a conclusion, we would like to stress that the brain is the organ with the second highest concentration in lipids, after adipose tissue, and that multiple brain diseases are linked to alterations in lipid metabolites. For instance, Alzheimer’s disease is tightly associated with cholesterol metabolism, both genetically and physiologically. [44], [45] Yet, surprisingly few studies were focused on adipose tissue as a source and storage for these key brain molecules. [46] Our current MRI study indicates that adipose tissue is also affected in its topography and potentially its function in ALS and calls for further functional studies on this key metabolic tissue.

## Subjects and Methods

### Patients

Sixty-two patients with ALS and 62 healthy volunteers underwent the MRI scanning protocol. The patients were recruited in the outpatient and inpatient settings of the Department of Neurology, University of Ulm, Germany. The age- and gender-matched control group without any neurological/psychiatric disease or other medical condition had been recruited through a volunteer panel (University for the Aged, volunteer work exhibition) or spouses of patients. Controls with neurological or psychiatric diseases or contraindications for MRI scanning were excluded from the study. All patients were diagnosed with definite (23) or probable (39) ALS using the El Escorial Criteria and had a disease duration of 22.55±15.25 months. Patients with gastrostomy, reduced respiratory function (FVC<60%) as well as patients with contraindications for MRI scanning were not included. The patients underwent a neurological interview to investigate the age, disease duration and the presence of bulbar symptoms. The information on diabetes mellitus and dyslipidemia were based on patient history, no laboratory data for these conditions were acquired in the study. One patient had diabetes mellitus, six patients had hyper-lipidemia. None of the volunteers had a history of diabetes mellitus, dyslipidemia or insulin resistance. Height and weight were measured in order to calculate the BMI. In addition, the technique of bioelectrical impedance measurement was used to determine the body composition. [13] A written informed consent was obtained from all participants. The ethical review committee of Ulm University approved this study (# 179/2008).

### MRI Measurements

MRI data were acquired on a 1.5 T scanner (Symphony, Siemens Medical, Erlangen, Germany). Bore size was 60 cm, horizontal. A quantum gradient system with gradient field strength up to 30 mT/m (52 mT/m effective) was available with a slew rate up to 125 T/m/s (216 T/m/s effective). The whole body MRI scan was recorded by acquisition of 6 to 8 T1-weighted volumes (standard T1 weighted spin-echo sequence), each consisting of 36 2-D slices (slice thickness 6 mm). In-plane resolution was 1.25 mm×1.25 mm (294×384 voxels), phase encoding direction was right to left, 206 phase encoding steps were used, zero-filling for image reconstruction. Slices were recorded with no gap. Flip angle was 20°, repetition time was 476 ms, and time to echo was 12 ms. The total acquisition time for one volume was 4∶30 minutes. To confirm that no gap is left in between the consecutive volumes, an overlap of about 6 to 18 millimetres was chosen between the volumes so that a total area of about 120 cm was scanned. [19].

### Data Pre-processing and Analysis

Data pre-processing was performed by the in-house developed software package ATLAS (Automatic Tissue Labelling Analysis Software). [19] The pre-processing consisted of several steps.

#### Registration - merging of the single volumes

Each of the single recorded 3-D volumes covered a body section of about 22 cm. Depending on the subjects’ heights, 6 to 8 volumes of this size were recorded. In between two consecutively recorded 3D-volumes, an overlap of between 6 and 18 mm, i.e. 1 to 3 slices, allowed for the application of a conjugated simplex fitting method with 3 degrees of freedom (x, y, z - translational shift). That way, one contiguous body volume was merged. Prior to subsequent analysis, the data were supersampled to isotropic voxels with size 1.2×1.2×1.2 mm^3^.

#### Data preparation: Intensity homogenisation and removing of arms

In cases that magnetic field or gradient inhomogeneities or distortions might cause image inhomogeneities over the large scanning area, an interactive repair functionality was used in order to correct the distortions and prepare the data set for further data operations. As the arms where often only partially recorded and were not of interest for further analysis, the arms were manually deleted from the data sets.

#### Intensity homogenisation: diffusion filtering

In order to homogenize intensity in the data sets, diffusion filtering was applied. [19].

#### Analysis

Subcutaneous fat determination was performed using the ARTIS algorithm (Adapted Rendering for Tissue Intensity Segmentation) which has already proven to reveal high stability in the results. [19] Abdominal visceral fat tissue was identified by selecting all connected voxels with respect to their intensity within the from ARTIS predefined range. [19].

### Statistical Analysis

Statistical analysis was conducted using Graphpad Prism 5 (GraphPad Software, La Jolla, USA). Since fat distribution in both patients and volunteers failed normality test according to D’Agostino and Pearson tests, we used the Mann-Whitney test for group comparisons. The Fisher’s exact test was used for dichotomic variables, and the Spearman correlation test for correlations. A multivariate regression analysis was performed/conducted using SPSS Statistics 19 (IBM, New York, USA). Log rank (Mantel-Cox) test was used to assess the effect of the different variables on survival. At the end of the study, 34 ALS patients had died and 23 were still alive. Patients still alive were censored for survival analysis. Significance was set at p<0.05.

## Supporting Information

Figure S1 **Total fat content in MRI scans correlates with surrogate markers of adipose tissue.** Correlations among total fat content (FC_MRI_), and bodymass index (BMI, **A**), fat content in bio-impedance metry (**B**) or circulating leptin (**C**). *p* values and the corresponding correlation coefficients (*r*) are indicated. If the correlation coefficient is positive, the two variables tend to increase or decrease together.
(TIF)
