# Supplementary figures and images for: Adipose Tissue Distribution Predicts Survival in Amyotrophic Lateral Sclerosis
Source: PLoS One. 2013 Jun 27;8(6):e67783. doi: 10.1371/journal.pone.0067783 (PMC3694869; doi:10.1371/journal.pone.0067783)

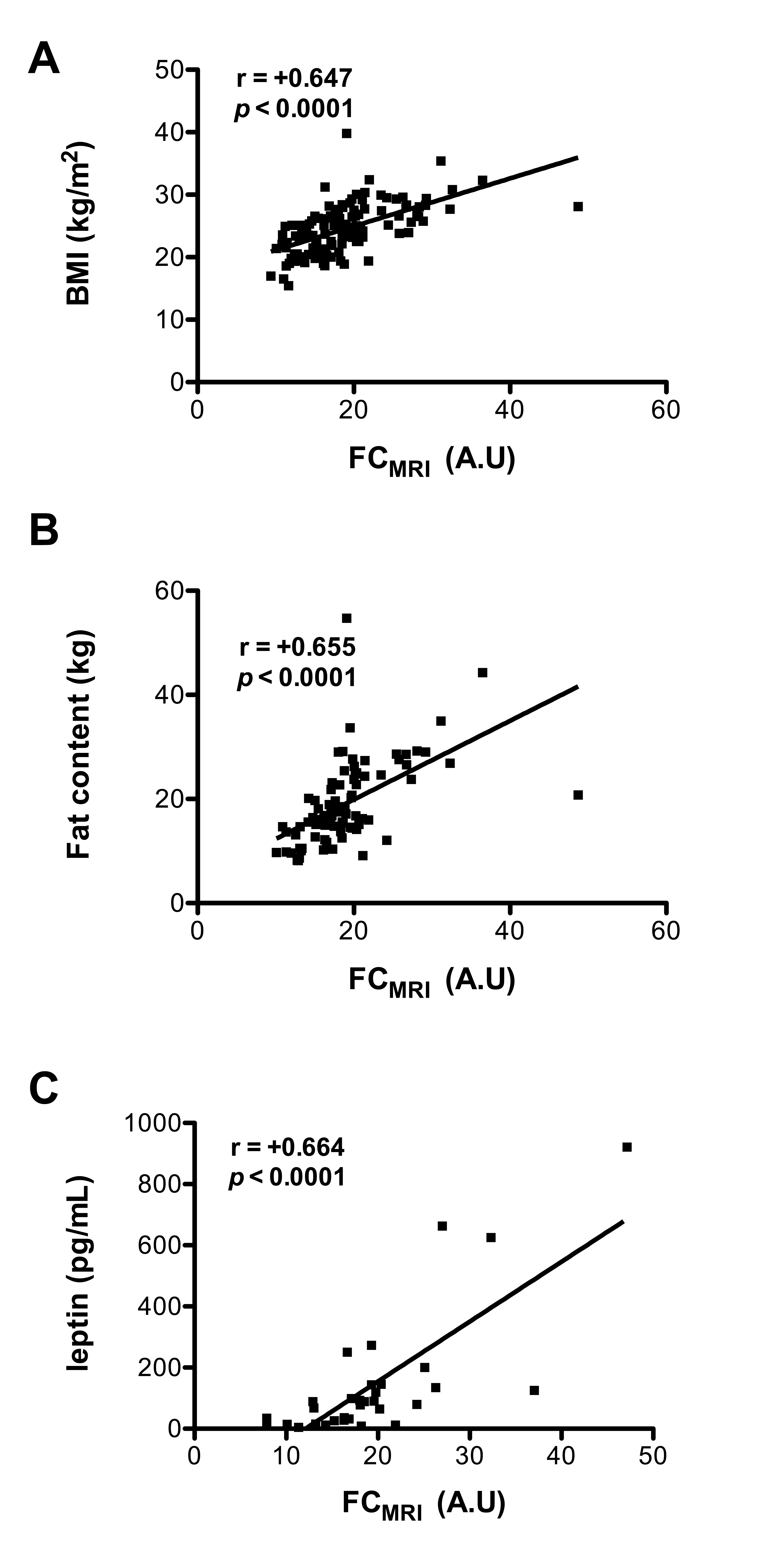

Supplement: Figure S1 — Total fat content in MRI scans correlates with surrogate markers of adipose tissue. Correlations among total fat content (FCMRI), and bodymass index (BMI, A), fat content in bio-impedance metry (B) or circulating leptin (C). p values and the corresponding correlation coefficients (r) are indicated. If the correlation coefficient is positive, the two variables tend to increase or decrease together. (TIF) [file pone.0067783.s001.tif]
